# Hybrid Vibration and UV Fluorescence Technology for Rapid Imaging and Guidance for Manual Removal of Fish Bones from Fish Floss

**DOI:** 10.3390/s22228978

**Published:** 2022-11-20

**Authors:** Yen-Hsiang Wang, Kuan-Chieh Lee, Wen-Chun Wei, Chung-Huang Wang, Hao-Jie Liu, Jia-Rong Hou, Tien-Chen Hsieh, Ju-Kai Chen, Ting-Yuan Chen, Shien-Kuei Liaw, Choa-Feng Lin, Chin-Cheng Wu, Jen-Jie Chieh, Chin-Hung Chang

**Affiliations:** 1 Institute of Electro-Optical Engineering, National Taiwan Normal University, Taipei 116, Taiwan; 2 Product and Process Research Center, Food Industry Research and Development Institute, Hsinchu 300, Taiwan; 3 Department of Electronic and Computer Engineering, National Taiwan University of Science and Technology, Taipei 106, Taiwan; 4 Department of Electronic Engineering, Asia Eastern University of Science and Technology, New Taipei 220, Taiwan; 5 Chemical Systems Research Division, National Chung-Shan Institute of Science & Technology, Taoyuan 325, Taiwan

**Keywords:** food safety, fish bones, fish floss, fluorescence, vibration, imaging, ultraviolet, monitor

## Abstract

The objective of the proposed human–machine cooperation (HMC) workstation is to both rapidly detect calcium-based fish bones in masses of minced fish floss and visually guide operators in approaching and removing the detected fish bones by hand based on the detection of fingernails or plastic-based gloves. Because vibration is a separation mechanism that can prevent absorption or scattering in thick fish floss for UV fluorescence detection, the design of the HMC workstation included a vibration unit together with an optical box and display screens. The system was tested with commonly used fish (swordfish, salmon, tuna, and cod) representing various cooking conditions (raw meat, steam-cooked meat, and fish floss), their bones, and contaminating materials such as derived from gloves made of various types of plastic (polyvinylchloride, emulsion, and rubber) commonly used in the removal of fish bones. These aspects were each investigated using the spectrum analyzer and the optical box to obtain and analyze the fluorescence spectra and images. The filter was mounted on a charge-coupled device, and its transmission-wavelength window was based on the characteristic band for fish bones observed in the spectra. Gray-level AI algorithm was utilized to generate white marker rectangles. The vibration unit supports two mechanisms of air and downstream separation to improve the imaging screening of fish bones inside the considerable flow of fish floss. Notably, under 310 nm ultraviolet B (UVB) excitation, the fluorescence peaks of the raw fillets, steam-cooked meat, and fish floss were observed at for bands at longer wavelengths (500–600 nm), whereas those of the calcium and plastic materials occurred in shorter wavelength bands (400–500 nm). Perfect accuracy of 100% was achieved with the detection of 20 fish bones in 2 kg of fish floss, and the long test time of around 10–12 min results from the manual removal of these fish bones.

## 1. Introduction

The ingestion of fish bones causes pain or discomfort in humans and is particularly dangerous for children and the elderly. Manufacturers may face problems when fish bones are present in processed food products, such as reputational damage and the need to refund customers. Hence, a suitable technology for the detection of fish bones in processed food products is urgently needed. Although X-ray instruments can detect metal objects in food or large bones in animal bodies on the basis of imaging contrast, they fail to identify fish bones at considerably lower densities inside food or metal foreign bodies within similarly shaped or sized food. Fish bone perforation can only be accurately identified in abdominal tissue using advanced computer tomography (CT), which displays this type of tissue with high contrast and involves a high degree of observer supervision. Similarly, fish bones lodged in the throat can only be detected using endoscopy [1,2]. Imaging technologies such as X-ray instruments, CT, and endoscopies are classified as structural imaging technologies.

In the food industry, X-ray imaging is limited to fish samples with thin and raw fish fillets and calcified bones. For example, fish bones in thin fish fillets (smaller than 2 mm) of salmon and trout can be examined in an X-ray examination room [3]. A commercial human–machine cooperation (HMC) machine (SensorX Fish, Marel, Iceland) can be used to identify calcified bones, metals, and stones in fish fillets, which are manually placed on and removed from a conveyor belt. The imaging mechanism relies on differences in the density and shape of dense foreign bodies vs. a thin meat fillet. Hence, this X-ray machine can detect fish bones only in fresh or thawed white fish, such as cods, haddocks, and hakes, but cannot in similar-sized minced pork or fish floss because they are side dishes served with rice and made by stir-frying steam-cooked shreds of meat. In addition, a significant quantity of fish floss products is transported to factories with other types of food, where they are used in several extension processes, such as the stuffing of eggrolls or bread. Hence, inspection for fish bone or pieces of broken plastic from packaging is necessary in food factories. Furthermore, precise guidance for manual and precise removal of the detected foreign bodies from the fish floss is also essential to reduce product waste. Guidance in the form of images on the display screen enables operators when moving their hands, with or without plastic gloves, to approach the target materials. Structural imaging, such as visual photos or X-ray images, cannot be used to guide the manual removal of fish bones inside large amounts of fish floss because of their small weight and similar appearance, so food safety in screening fish bones from floss typically relies on human input with reliance on senses, such as visual inspection and hand touch, rather than any type of inspection technology. However, these human senses are both inefficient and unreliable in detection.

Different from X-ray structural imaging methods, a functional imaging method based on the ultraviolet (UV)-excited fluorescent imaging of fish bones, which are mainly composed of calcium carbonate [4], can be achieved via the mechanism of UV-induced fluorescence detection of calcium-based materials [5]. For example, coronary artery calcification can be identified by detecting the main component of tricalcium phosphate polymerization using UV-induced fluorescence spectroscopy [6]. UVA light has been used to detect bones in raw chicken meat according to the calculated fluorescence intensity ratios of 510, 575, and 620 nm [7]. Similarly, a UVA lamp has been utilized in the fluorescent imaging of fish bones in ultrathin raw cod fillets [8]. Actually, the identification of fish bones in the shallow distribution of the cod fillets could be made by human vision, without the need of any instruments. Additionally, UV-induced fluorescence spectroscopy has been applied to determine the freshness index of frozen foods and surface cooking conditions in raw and cooked meat, for example [9,10]. Hence, the use of UV-induced fluorescence to detect fish bones in cooked meat or fish floss remains challenging and is as yet unsuitable for industrial application.

Notably, the detection performance of UV-induced fluorescence is limited in two main aspects. One is the lack of a suitable excitation wavelength for the fluorescence discrimination of fish bones from any fish meat. The other is depth, which is a well-known barrier to fluorescence propagation because of its low penetration ability. The ability of fluorescence to discriminate between fish bones and fish meat can be improved by performing a complete optical study. The use of fluorescence detection has always been limited to shallow target materials because the scattering or absorption of the excitation or fluorescent light increase with the thickness of the covering materials. This problem could be solved for minced materials by auxiliary separation mechanisms, which are used in some industry transportation processes. For example, a separation mechanism involving the use of air vibration can be used to temporarily separate the target and cover materials in air, subsequently allowing light to be efficiently propagated through this space. The target material and other materials can then be separated downstream, i.e., after the vibration process. Structural imaging, such as X-ray or visible inspection, depends on the high contrast of nonspecific foreign bodies in thin and transparent flakes, such as raw white fish fillets, during horizontal transportation on common conveyors. Hence, these current structural imaging methods are unsuitable for fish bones deeply embedded within dark-colored fish floss. In contrast, in the vibration unit of the proposed HMC workstation, a tilt plate and vibration motor separately supported the horizontal transportation and the separation mechanisms. Separation of the minced materials assists the functional imaging of UV-induced fluorescence and allows the depth limitation of fish bones within fish floss to be overcome.

In other words, vibration and UV-induced fluorescence technologies are hybridized in the system developed and proposed in our study, and this system should not only enable the detection of fish bones deep inside fish floss but also guide in the manual and precise removal of materials by hand based on the detection of plastic gloves or human fingernails. To achieve this goal in this study, we first used a spectrum analyzer to determine the fluorescence profiles of different samples of fish meat (with variation according to cooking) of commonly used fish species and some widely used types of plastic gloves. In addition, an HMC workstation based on UV-induced fluorescence, artificial intelligence (AI) algorithms, and vibration was developed to achieve rapid low-budget screening and exclusion guidance for use in the food industry.

## 2. Experimental Section

**Figure 1:**
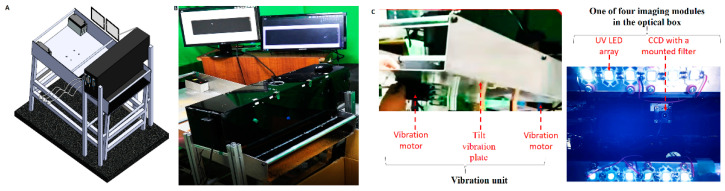
Proposed HMC workstation consisting of an optical box with black shielding, a vibration unit, and two monitors: (**A**) schematic and (**B**) photograph. (**C**) Photos of a vibration unit and an imaging module inside the optical box.

The proposed HMC workstation (Figure 1A,B) comprised an optical box, a vibration unit (Figure 1C), and two monitors. The optical box, a black box eliminating environmental light with an open bottom for UV illumination and imaging, is the first component of the proposed workstation and contains an imaging line array and electronic equipment, including a microcontrol unit (MCU). Here, four imaging modules arranged at intervals of 8.5 cm constitute the imaging line array (right-hand side of Figure 1C). Each imaging module consists of a charge-coupled device (CCD) (CCD 700, Sony Corporation, Kanagawa, Japan), 310 nm UV light-emitting diodes (LEDs) (3535 UVB LED, Galaxy Scientific Equipments, Dombivli, India), and an optical band-pass filter with a peak wavelength of 405 nm and full width at half maximum of 10 nm, which was manufactured by the Taiwan Instrument Research Institute (TIRI).

**Figure 2:**
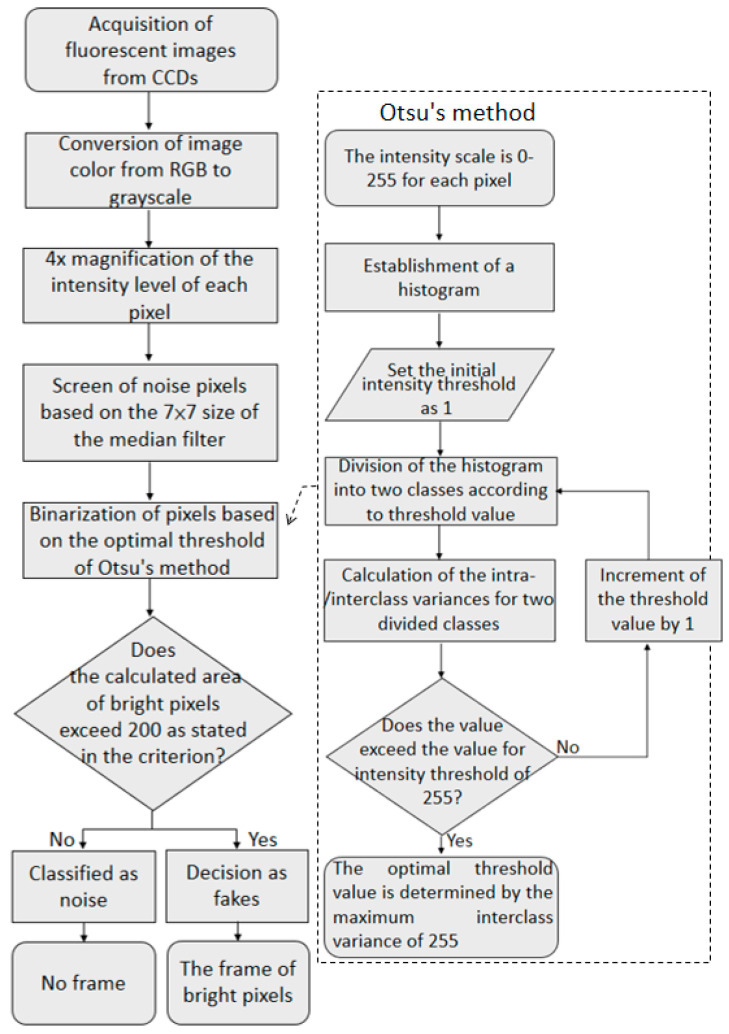
AI algorithm program for the generation of white rectangular markers of fish bones based on the fluorescent intensity of CCD pixels.

The electronic devices contained a microcontroller unit (MCU) (Jetson Nano, NVIDIA Corporation, Santa Clara, CA, USA). The following AI image algorithm process was utilized for each CCD frame. Each frame was converted to grayscale, and the intensities of all pixels were adjusted to four times their original levels in a linear manner to enhance the fluorescent signal and compensate for the short exposure time. On the basis of the threshold selection method from the gray level [11,12], the weak fluorescent intensity of the fish floss acted as the noise for comparison with the strong fluorescent intensity of fish bones or plastic gloves. Fish bones or plastic gloves and their white marker rectangles were separately examined. For example, the pixel number of the bright region was more than 200 (Figure 2). In addition to the algorithm for the white marker rectangle, the on–off state of vibration motors was controlled using the MCU.

The vibration unit (left-hand side of Figure 1C) forms the second part of the HMC workstation and is composed of two vibration motors, four springs, and a large tilt plate (70 cm (width) × 120 cm (length) × 75 cm (height of the middle part)) with a tilt angle of 9°. Vibration motors (B-402, Chuan Sheng Electric Co. Ltd., Taoyuan, Taiwan) were attached at the bottom of the upper and lower parts of the tilt plate. The optical box was placed above the end region of the tilt plate because of the high static separation at the end of this plate. A black cutting sheet made of food-grade vinyl was pasted onto the tilt plate under the optical box to avoid background light scattering, although at the expense of fish bone fluorescence, to some extent. Two monitors (SA300, Samsung, Suwon, Korea) were used in the third part of the HMC workstation to guide the hand with or without plastic gloves for the manual removal of detected fish bones (Figure 1A,B).

### 2.1. Workstation Test

**Figure 3:**
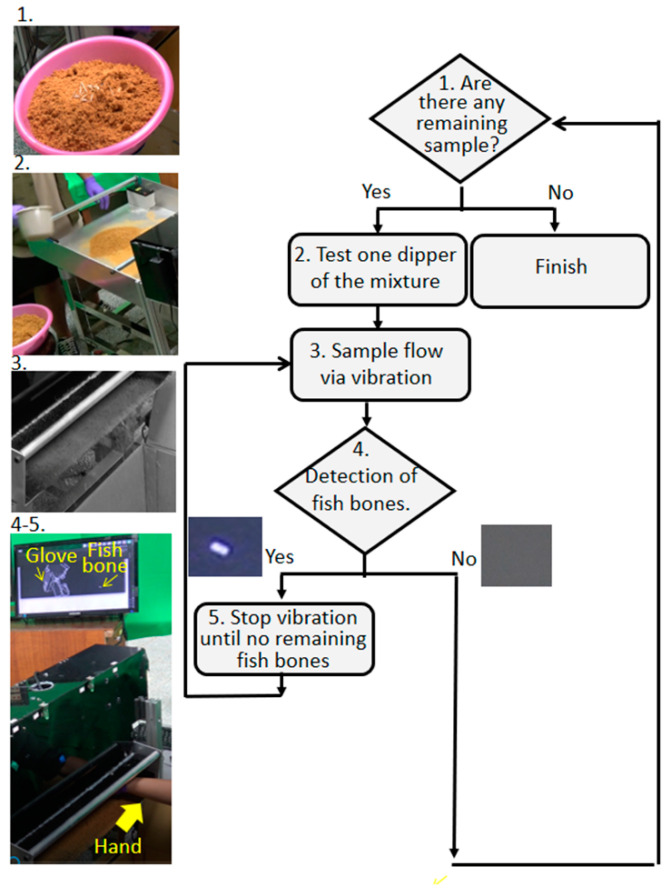
Control program for performance tests.

On the basis of industry throughput, a mixture of 20 fish bones and 2 kg of fish floss (step 1, inset of Figure 3) was tested 10 times to verify the capabilities of vibration transportation, fish bone imaging, and manual exclusion guidance. One dipper (300 mL) of this mixture was poured onto the tilt vibration plate (inset of step 2 in Figure 3). If fish bones were absent in imaging under the optical box, then the continuous vibration allowed this mixture to flow away from the end of the tilt vibration plate (step 2, inset of Figure 3). Conversely, vibration was immediately stopped if fish bones were present under the optical box as determined based on the imaging in air or on the tilt vibration plate (step 3, inset of Figure 3). The sample corresponding to the imaged fish bones would remain on the tilt vibration plate while completely covered with fish floss (step 4, inset of Figure 3). Whole plastic gloves and other white moving markers were used for guidance in approaching the original and static white markers on the monitors (step 5, inset of Figure 3). The vibration automatically restarts when the white markers, such as fish bones and gloves, disappear. It was noted that the repetition interval before the next scoop of mixture was around 1 min after the amount remaining from the previous mixture had disappeared. Each round of processing of 2 kg of fish floss lasts around 10–12 min.

### 2.2. Materials and Spectrum Studies

Test materials included fish bones, raw meat, steam-cooked meat, and fish floss of four commonly used fish types: salmon, swordfish, tuna, and cod as well as commercial fish floss of salmon, cod, tuna (Wei-I Foodstuff Co., Ltd., Pingtung, Taiwan), and swordfish (Hsin-Tung Yang Co., Ltd., Taoyuan, Taiwan). Fish bone samples were 1–10 and 5–20 mm in width and length, respectively. Fillet dimensions were around 15 mm × 15 mm × 5 mm. The size of fish floss pieces was around 1–3 mm.

On the HMC workstation, fish bones were manually removed by human hand with or without common plastic gloves. Hence, powders of calcium carbonate (325 mesh, Emperor Chemical Co., Ltd., Taipei, Taiwan) were utilized as proxies for fingernails or fish bones [13,14]. Three commonly used plastic gloves of different materials, namely, rubber (H1167 nitrile examination glove, Well-Pack Industries Co., Ltd., Taipei, Taiwan), polyvinyl chloride (PVC) (Maslee Patient Examination Gloves, Sun-max Vietnam Co., Ltd., Haiphong, Vietnam), and emulsion (emu) (H511 powder free examination gloves, Haw Ping Co, Ltd., Changhua, Taiwan) were tested because of their fluorescence [15].

**Figure 4:**
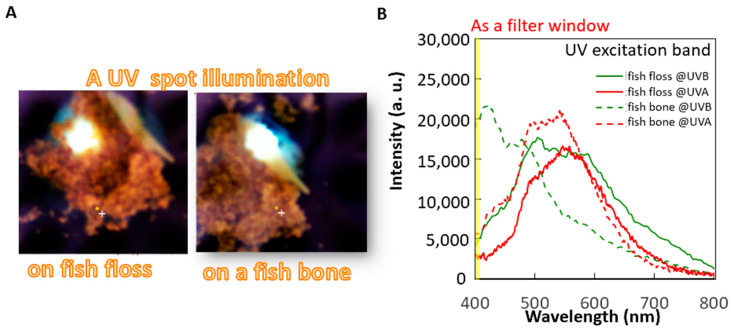
Spectrum analysis of individual fish floss and bone under UVA (365 nm) and UVB (310 nm) excitation: (**A**) photos of the separation illumination and (**B**) fluorescent spectra.

**Figure 5:**
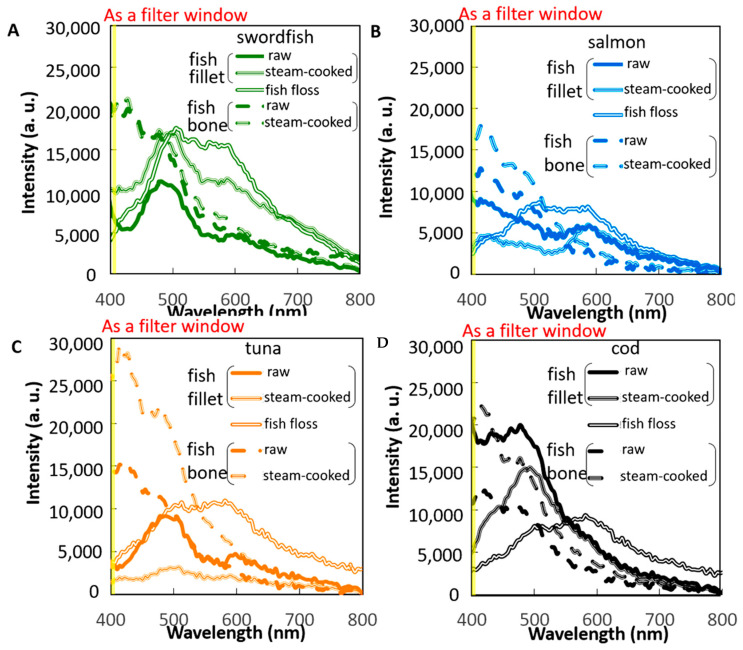
Fluorescent spectrum of fish meat and its fish bone under different cooking conditions (raw, steam cooked, and fish floss) corresponding to the four fish types of (**A**) swordfish, (**B**) salmon, (**C**) tuna, and (**D**) cod.

**Figure 6:**
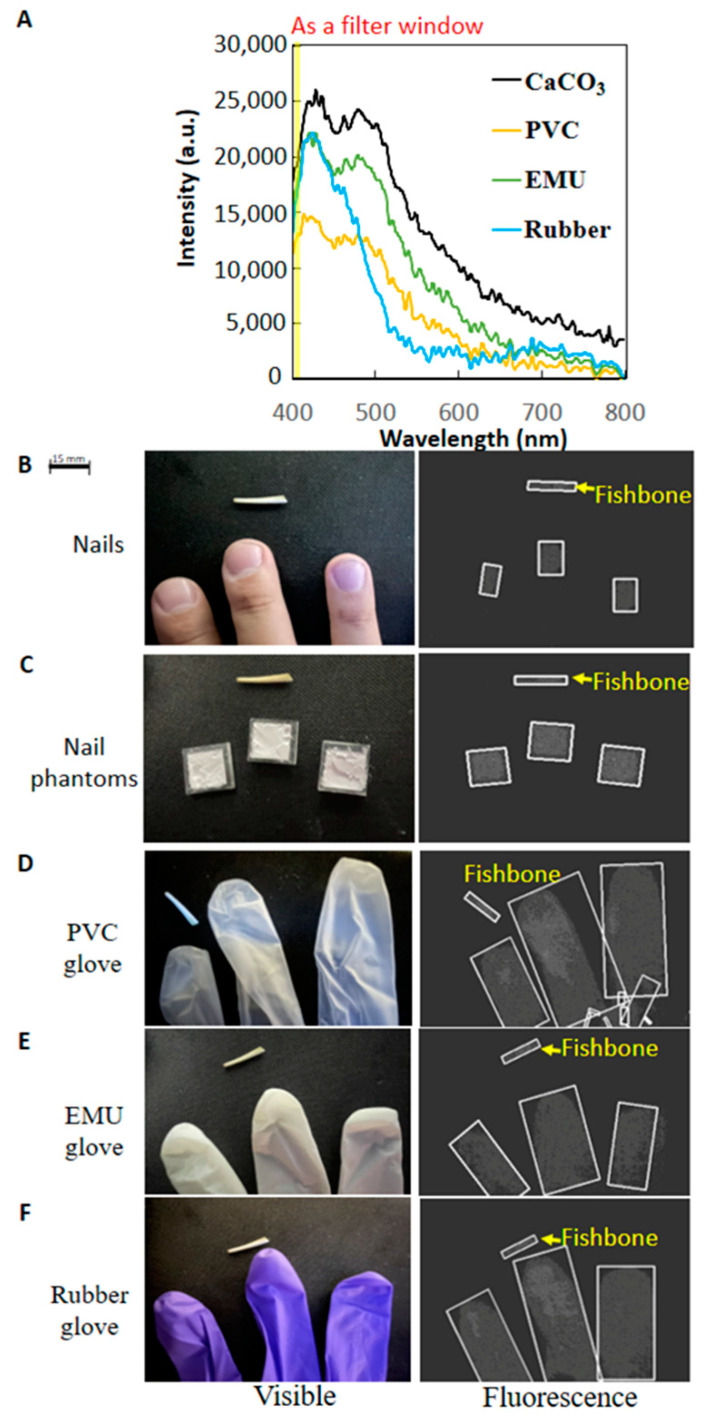
UVA (310 nm)-induced fluorescence spectra and images of exclusion materials also in the form of powder. (**A**) Fluorescent spectra of powders of calcium carbonate and three plastic types (PVC, EMU, and rubber) of commonly used gloves. Fluorescent image of a fish bone as the exclusion target and five samples of (**B**) human fingernails, (**C**) phantom fingernails of calcium carbonate powders, (**D**) fingers of a PVC glove, (**E**) fingers of an EMU glove, and (**F**) fingers of a rubber glove.

**Figure 7:**
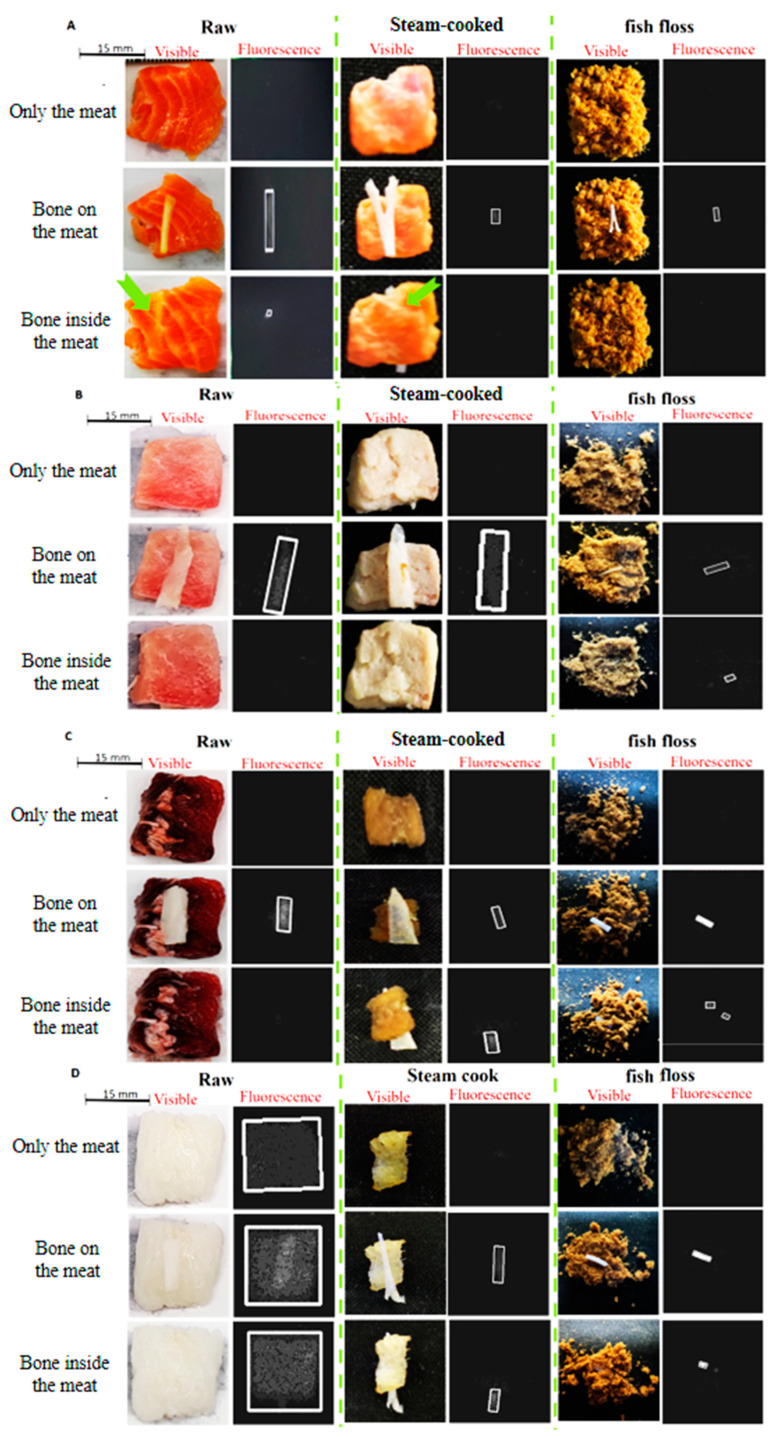
Visible and fluorescence images of fish meat and its bone under different cooking conditions (raw meat, steam-cooked meat, and fish floss) and arrangements (meat only and a fish bone over or inside its meat or fish floss). The four fish types were (**A**) swordfish, (**B**) salmon, (**C**) tuna, and (**D**) cod.

**Figure 8:**
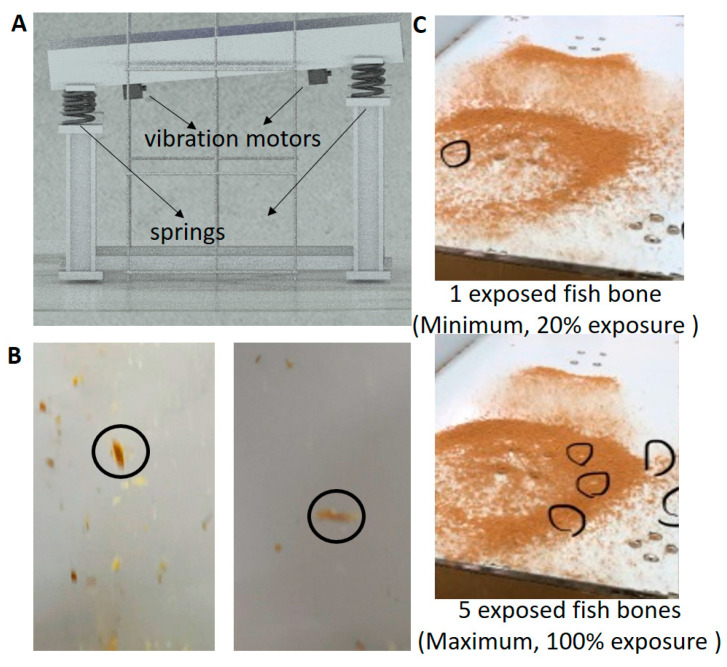
Two separation mechanisms of the vibration unit: (**A**) the unit scheme, and the mechanisms of (**B**) air separation, and (**C**) downstream separation. The tested mixture was composed of five fish bones and 500 g of fish floss.

The UV-induced fluorescence of these materials was characterized in three steps. According to the spectrum analysis (OS361AC55007649, OtO Photonics Inc., Hsinchu, Taiwan) and 365 (LG 3535, Cree Inc., Durham, NC, USA) and 310 (3535 UVB light-emitting diodes (LEDs), Galaxy Scientific Equipments, Dombivli, India) nm wavelength types of UV LEDs, the UV band suitable for fluorescence testing that allowed clearly discriminating between the cooked fish bone and the fish floss of the most popular swordfish in the market was confirmed in the first step (Figure 4). The second step was to confirm whether the specification determined in the first step was workable for the various cooking conditions (raw meat, steam-cooked meat, and fish floss), the four commonly used forms of fish (Figure 5) and the exclusion materials (Figure 6A). The average acquisition time of the spectrum analyzer was 30 ms. In the third step, the imaging module of the proposed HMC workstation was validated for the imaging of fish bones alone (Figure 7), its different arrangements for mixed fillets or fish floss, and in approaching exclusion materials (Figure 6B–F). Here, the imaging results (Figure 6B–F and Figure 7) were derived from static sample arrangement under the optical box rather than the workstation test of vibrating samples in dynamic flow (Figure 8).

## 3. Results

A small spot of UV excitation was separately projected onto these materials for the spectrum analysis of fish bones and meat under different cooking conditions (raw meat, steam-cooked meat, and fish floss). The first analysis aimed to determine the optimal excitation choice between UVA (365 nm) or UVB (310 nm) bands using the fluorescence spectra of the most commonly used fish floss of swordfish on the market and its bones (Figure 4B). Both the fish bone and floss generally expressed the same fluorescence profile in the visible band (400–800 nm), i.e., a peak at 560 nm, under UVA of 365 nm excitation. However, under a UVB excitation of 310 nm, the fluorescent peak of the fish bone occurred at a shorter wavelength of around 410 nm, but that of the fish floss merely slightly broadened the same peak of the fluorescence profile, similar to the peak for UVA excitation. The fluorescence profile of fish bones and floss differed under 310 nm but remained the same under a UV excitation of 365 nm. Hence, 310 nm UV LEDs were used as linear UV LED imaging module arrays in this study to distinguish the fish floss from fish bones (Figure 1C). Furthermore, the maximum difference in intensity occurred in the wavelength duration of 400–410 nm and is depicted in yellow by the filter mounted on the CCD (Figure 4B and Figure 5A–D).

Spectrum analyses of the four commonly used fish meats with an excitation wavelength of 310 nm under different cooking conditions (raw meat, steam-cooked meat, and fish floss) and their fish bones were explored to evaluate their suitability for use in the fish industry (Figure 5). All cooking conditions of three widely utilized fish meats (swordfish, salmon, and tuna) demonstrated a similar fluorescence profile, consisting of a decreased slope or a peak far from the filter window of a yellow label (Figure 5D). It was noted that the fluorescence intensity levels of raw or cooked meats in the short wavelength were seldom higher than the threshold value of 10,000 in the criterion for abnormality. By contrast, fluorescence intensities for raw or cooked fish bones were higher than this threshold value for abnormality (Figure 5). The white marker in the fluorescent images from a filter-mounted CCD was generated from the differences in fluorescence intensity using the AI algorithm program (Figure 2 and Figure 7).

The same fish bones were arranged under different assembly conditions (none, and fish bones on or inside the raw meat, steam-cooked meat, or fish floss) to determine the feasibility of risk detection using the proposed HMC workstation (Figure 7). No white marker was generated for fish meat alone under all cooking conditions (Figure 7A–C), with the exception of the raw cod (Figure 7D). A white marker filled with light or intense white spots was observed in all the fish bones within the meat (Figure 7) and in some exposed parts of fish bones inside the fish floss or steam-cooked meat (Figure 7B–D). Conversely, white markers were absent for fish bones inside steam-cooked meat (Figure 7A,B) and fish floss.

Because the detection results correspond to samples of partially exposed fish bones, the investigation using the proposed HMC workstation only focused on minced food, such as fish floss, rather than raw or cooked fillets (Figure 7). Two vibration motors under the rear side of the tilt plate and four sets of springs on four feet were designed to generate and reduce the vibration, respectively (Figure 8A). The main component of the tilt vibration supported the separation mechanisms to increase the opportunities to expose fish bones from fish floss, and the test mixture of five fish bones and 500 g of fish floss, that is, one-quarter of the total workstation test amount, was used in four separate tests. The two observed separation mechanisms were separation in air (Figure 8B) and downstream of the tilt vibration plate (Figure 8C), where black circles indicate the exposed fish bones. The separation in air exposed all the fish bones in the four tests. On the other hand, the number of fish bones exposed in the mechanism downstream of the tilt vibration plate varied. For example, one to five fish bones were exposed downstream in each of the four tests. Completely covered fish bones were seldom exposed.

Although the exposed fish bones were detectable, the proposed HMC workstation should be used as a guide for the manual exclusion work. In the same way as for the fish bone or different cooking methods and fish types, the fluorescence spectra of exclusion materials and chemical composition powders of the same sizes of 15 mm × 15 mm were examined (Figure 6A).

Powders of calcium carbonate exhibited significantly higher intensity fluorescence than those of the three types of plastic (PVC, EMU, and rubber) of widely used gloves because of their common fluorescence peak close to the right-hand boundary of the yellow-labeled filter window. Consequently, white marker rectangles clearly highlighted for exclusion three human fingernails and three phantom fingernails with the same fishbone in fluorescent images (Figure 6B,C). Furthermore, fingers clad in commonly utilized plastic gloves, i.e., PVC, EMU, and rubber, were also imaged as three large white marker rectangles for exclusion (Figure 6D–F), possibly accompanied by some small rectangles. Finally, an accuracy of 100% was achieved in 10 workstation tests with a mixture of 2 kg of fish floss and 20 fish bones (Figure 3) at around 10–12 min per test.

## 4. Discussion

Risk materials or foreign bodies of low density in fish meat, such as fish bones or minced plastic packaging, remained undetected when using X-ray-based technologies but were successfully detected using the proposed HMC workstation based on UV-induced fluorescence (Figure 6B–F and Figure 7). This result can be explained according to the following considerations.

The first is the UV excitation method for differentiating between fish bones and floss. The 310 nm UV light in the UVB band (280–320 nm) has higher photon energy than the common UVA band (320–400 nm) in inducing a shorter wavelength fluorescence range (400–500 nm) for some special components, such as calcium-based fish bones and fingernails (Figure 4B) [13,14]. In addition, a UVA-induced fluorescence peak in the longer wavelength range (500–600 nm) is commonly observed (Figure 5 and Figure 6A), particularly for calcium-based materials.

Calcium concentrations in food that are higher or lower than the criterion of 6 mg in 100 g of meat can be easily determined from the fluorescent peak of the tested food under UVB excitation. Fluorescent peaks of samples with lower calcium concentrations, such as 4 and 5 mg in 100 g of swordfish and tuna meat [15], under any cooking condition (raw meat, steam-cooked meat, and fish floss) persist in the longer wavelength range of 500–600 nm (Figure 5A,C). However, the fluorescent peaks of raw fillets with higher calcium concentrations, such as 7 and 16 mg in 100 g of salmon and cod meat [15], respectively, are observed in the shorter wavelength range (400–500 nm), whereas those of steam-cooked meat and fish floss are observed in the longer wavelength range (500–600 nm) (Figure 5B,D). Calcium loss always occurred after the meat was cooked, particularly when sauces contained sodium [16]. The increase in fluorescence intensity in the short wavelength range (400–500 nm) as calcium concentrations increase is consistent with other calcium ion indicators [12].

Consequently, fluorescence intensity in the feature spectrum band of 405 nm in the center and 10 mm in width was used as the yellow-labeled filter window of the adopted CCD. Although the fluorescence intensity was higher than the criterion of, the white biomarker rectangles were roughly generated, using the AI algorithm, as the calcium distribution labels in the food. For example, calcium carbonate-rich fish bones under all cooking conditions (Figure 5) had a concentration of 15–26% calcium by weight [17,18,19]. A raw cod fillet (Figure 5D) and calcium carbonate powders of a phantom fingernail (Figure 6A) presented a calcium weight concentration of 16% and 39.2%, respectively. In particular, the sensitivity of the white biomarker rectangle was sufficiently high for human fingernails (Figure 6B) at 0.1%, i.e., 1000 ppm [20] and the no-calcium exceptions of the three commonly applied PVC, EMU, and rubber materials because of their good UV absorption [17]. Consequently, the shapes of the white marker rectangles in the fluorescent images of the high calcium concentration or plastic samples (Figure 7 and Figure 6C–F) were close to the true shapes. In addition, white marker rectangles were absent from the fluorescent images (Figure 7) of fish meat under all cooking conditions, with the exception of raw cod, because the fluorescent intensity in the yellow-labeled feature spectrum was below the criterion. The criterion for the generation of white markers was set at a value that was low enough to allow both fish bones and fingernails to be imaged at high and low calcium concentrations, respectively, in addition to common plastic materials. Furthermore, based on the shapes and appearance conditions (dynamics and statics) of the fluorescence image, the operator could easily identify fillets (as static rectangles in Figure 7D), plastic gloves (as a few moving and closing bars in Figure 6D–F), and fingernails (as a few moving and closing squares in Figure 6B).

The original broad visible range (400–700 nm) of pixels was limited with the attached filter to a narrow window (400–410 nm) to facilitate high specific detection of calcium- or plastic-based materials using the CCD. This constraint of a short detection wavelength band excludes not only the UV excitation light at 310 nm but also visible light from the surrounding environment. Hence, with the integration filter-mounted CCD and the black box against the environment light representing the imaging modules of the proposed HMC workstation, the sample fluorescence was the main contributor to the detection of visible-band light, with little sample reflection or light scatter. As a result, fluorescence images of high-specification materials were generated rather than structural images that have high dependency on component boundaries. The difference in the fluorescence spectra between fish bone and fish floss can be explained by Figure 4B. Generally, the spectrum profile of any detected light expresses one peak in a narrow characteristic wavelength band, and the spectrum profiles of the two slopes in the longer and shorter wavelengths tend to gradually decay compared with the band in this characteristic wavelength. In other words, different spectrum profiles commonly exist, but do not overlap, in the spectrum wavelengths, especially the different characteristic wavelength bands of their peaks in the narrow ranges, which are well known and the focus of spectroscopy for materials identification. Furthermore, the peak intensity can be also analyzed via the set intensity criterion using a simple and economic combination of the mounted optical filter with high transmission in the narrow characteristic wavelength band together with large-scale detection CCDs or point-detection optical detectors for summarizing total intensities in the narrow wavelength band in contrast to point-detection and high-cost spectrum analyzers with rich wavelength information. The reason for this is the fluorescence in the visible wavelengths band, and many mature manufacturing technologies are represented by optical devices that are compact, affordable, and readily available. Moreover, the analysis of the spectrum profiles can be solved using AI algorithm methods, such as principal component analysis [21].

Only the fluorescence image or so-called functional image was constructed using key compositions, such as calcium or plastics, under UVB illumination. Notably, although fluorescence technology is widely applied, the detection of fluorescent light is limited to shallow depths. For example, artificial fluorescence indicators inside superficial tissue are utilized for guidance in the surgery of lesions [20], and self-fluorescence of chlorophyll in orchid leaves is used in screening virus infection [21]. This feature is consistent with the white marker rectangles observed in fish bones inside raw salmon fillets (Figure 7A) but not in all fish types or fish fillets under different cooking conditions.

Therefore, the detection of target fluorescence materials can be more successfully achieved against the thick coverage of a minced meat than at shallow depths in any tissue, leaf, or fillet because of the vibration union in the HMC workstation. Furthermore, two vibration mechanisms of downstream and air separations were revealed in the tilt vibration plate. The phenomenon of downstream separation was similar to the separation of submerged objects moving at different velocities over very long distances [22]. Here, the density differs between the immersed objects (fish bones) and the flow materials (fish floss). Although the downstream separation was unrepeatable (Figure 8C), air separation in the vertical direction (Figure 8B) was the most crucial phenomenon for eliminating the thick coverage of fish floss on fish bones against the propagation barriers of fluorescent light. Moreover, air separation under the optical box was induced by the lower vibration motor under the rear side of the tilt vibration plate, and downstream separation was induced by the upper vibration motor (Figure 8A). Using air separation, many spaces between fish bones and fish floss were introduced for the illumination of UV light and the transmission of fluorescent light by vibration force. Hence, its efficiency or success could be evaluated by the parameter formula $\frac{I_{a}}{I_{0}}$, where I is the mean intensity of detected fluorescence from the tested fish bones, and the subscript “0” and “a” are represent the no cover and air separation conditions of the tested fish bones, respectively. In other words, the efficiency range was adapted to 0–1 by normalizing the detected fluorescence of the tested fish bones under the air separation condition to those under conditions of no cover. This indicates that the conditions for best or failed air separation contribute to the effectiveness of fluorescence detection of the tested fish bones as much as the conditions of “no cover” or “undetectable”. Evaluation of the suitability of the detection technology was determined based on differences in the properties of the target vs. normal materials or contained products rather than by just the target foreign body. For example, color differences are revealed by visual observation whereas X-ray technology is used to determine density and size differences, such as in the case of distinguishing small objects made of high-density metal or low-density plastics [23] and fish bones in uniform and low-density plate materials of hollow boxes or solid meat. General fluorescence technology can detect fluorescent materials, such as chlorophyll in leaves and fish bones in fillets. The HMC workstation based on vibration-assisted and UVB-induced fluorescence technology can be used to identify calcium or plastic foreign bodies in thick-coverage minced cooked fish meat (i.e., fish floss) based on the fluorescence difference in the narrow wavelength band (400–410 nm).

Structural imaging, such as visual and X-ray imaging, identifies differences in density and geometry, and complex image analysis via the AI algorithm uses expensive algorithm devices, complicated deep learning methods, and big data regarding shapes, sizes, and outer colors. Notably, highly specific consideration of material composition improves the ease of using functional imaging algorithms, such as machine learning.

Functional imaging based on UV-light illumination is similar to structural imaging based on visible-light illumination. Both these imaging methods are much safer than structural imaging based on X-ray illumination. The risks of radiation exposure to operators or products can sufficiently increase as a result of corrosion in passive shielding or nonfunctional active controls of the X-ray inspection machine. Furthermore, functional imaging based on UVB-light illumination may also be used to support UV light sterilization function [24,25] for food inspection and is safer for the manual removal of foreign bodies than methods based on UVC-light illumination.

Inspection accuracy using the optical box was 100% for all 10 tests. In addition to accurately detecting all the fish bones in the eight tests, in the two other tests, one detected fish bone disappeared immediately although the vibration motor was stopped. The missing fish bone was directly pushed from the tilt vibration plate into the collection box by the inertial force of vibration. Each test lasted approximately 10–12 min, during which the imaging inspection was as fast as a few milliseconds, although the manual removal under the guidance of the display took several minutes to complete. Clearly, costly automated exclusion methods could significantly expedite the inspection rate in the future. The imaging module of the proposed HMC workstation based on UVB-induced fluorescent technology to identify calcium- or plastic-related materials can detect fish bones without thick coverage and guide human fingernails or plastic gloves to remove fish bones. The tilt vibration plate separated the pieces of fish floss from the fish bones using two mechanisms, and satisfactory detection performance was achieved. The accuracy of the millisecond-rapid inspection was 100% for 20 fish bones in 2 kg of fish floss. The manual removal of these fish bones significantly prolonged the total time to 10–12 min.

For the sensing process, the combination of a standard CCD and mounted optical filter for the required large-scale detection and narrow fluorescence band was much less expensive than a spectrum analyzer for the unmet ultra-small detection range and the unnecessarily broad spectrum. Furthermore, the current size of the tilt plate was suitable for both the efficient vibration of piece-shaped products and the detection of the fluorescence of fish bones. The larger size of the tilt plate than the ones currently used generally reduced the effectiveness of the hybrid technique. Hence, for a large volume of inspections, the number of current HMC workstations could be fabricated massively, designed flexibly, and assembled for one multi-channel workstations, instead of one large-size HMC workstation.

## 5. Conclusions

In the beginning of this work, we utilized the expensive and limited-detection-size spectrum analyzer to study the UVB-induced fluorescence of two different pure material without any coverage: (1) calcium-related fish bone and meat under three cooking conditions using four commonly used fish types in addition to fingernails and (2) plastic-based gloves. Next, the integration of a filter-mounted CCD and an AI algorithm worked well to replace the aforementioned spectrum analyzer and enlarge the detection area but still failed to achieve adequate detection under conditions of coverage. However, via the two vibration mechanisms of the vibration union, the developed HMC workstation successfully achieved rapid and stable identification of fish bones with superior accuracy, even when thickly covered by a large mass of fish floss, enabling manual removal under the guidance provided by the display screen. Furthermore, the HMC workstation exhibits advantages in addition to conventionally used structural imaging approaches. The proposed HMC method can be used for the industrial detection of rich calcium- or plastic-based foreign bodies in masses of minced and low-calcium products.

## Data Availability

Not applicable.
